# Comparison of the Miniaci and Dugdale techniques on functional outcomes in medial open wedge high tibial osteotomy

**DOI:** 10.1186/s40634-023-00653-5

**Published:** 2023-08-24

**Authors:** Ümit Aygün, Murat Bölükbaşı, Kamil Yamak, Ali Can Çiçek

**Affiliations:** 1 Faculty of Medicine, Department of Orthopaedics and Traumatology, Ağrı İbrahim Çeçen University, Ağrı, Türkiye; 2 Department of Orthopaedics and Traumatology, VM Medical Park Samsun Hospital, Samsun, Türkiye; 3 Buca Seyfi Demirsoy Training and Research Hospital, Orthopedics and Traumatology Clinic, İzmir, Türkiye; 4 Ağrı Training and Research Hospital, Orthopaedics and Traumatology Clinic, Ağrı, Türkiye

**Keywords:** High tibial osteotomy, Medial open wedge, Correction angle, Miniaci technique, Dugdale technique, Measurement

## Abstract

**Purpose:**

To compare the correction angles determined by the Miniaci and Dugdale techniques in patients treated with medial open wedge high tibial osteotomy (MOWHTO) and show their impact on clinical outcomes.

**Methods:**

Seventy-four patients constituted the study group. The correction angles in Group 1 were measured using the Miniaci technique, and those in Group 2 were measured using the Dugdale technique. The clinical evaluations included the Tinetti Gait and Balance Assessment (TGBA), the Western Ontario and McMaster Universities Osteoarthritis (WOMAC) scores, and the Visual Analogue Scale (VAS). The effect of the correction angle on the patient's clinical outcomes was evaluated. Measurement techniques were also changed between groups for comparison.

**Results:**

Seventy-four patients (62 females, 12 males) with a mean age of 53.7 ± 4.9 years were followed up for a mean of 67.4 ± 5.5 months. The TGBA, WOMAC, and VAS scores were improved at the last follow-up compared to the preoperative scores (*p* < 0.05). The preoperative TGBA and WOMAC scores were not significantly different between the two groups, but the last follow-up TGBA and WOMAC scores in Group 2 were worse than those in Group 1 (*p* < 0.05). When measuring techniques were changed, the preoperative correction angle (PCA) value and the last follow-up correction angle (LFCA) value were lower in Group 1 measured with the Dugdale technique but higher in Group 2 measured with the Miniaci technique (*p* < 0.05).

**Conclusion:**

Since the correction angle values measured with the Miniaci technique in MOWHTO are higher than those measured with the Dugdale technique; the functional results are better.

**Level of evidence:**

Retrospective cohort study, III.

## Background

In high tibial osteotomies, correcting the weight-bearing axis is the basis of the surgical plan [7]. A new mechanical axis is important to achieve better clinical outcomes. The exact location at which the axis passes through the knee region remains unknown [6]. In many studies, researchers have suggested that achieving optimal correction of the mechanical axis is challenging because only 70–80% of the axis is within the desired range postoperatively [17, 42].

It has been shown that the mechanical axis in mild valgus promotes cartilage healing and produces better functional outcomes [8, 15]. Thus, although the Mikulicz point crosses slightly lateral to the knee joint and can be used to determine the proper long leg mechanical axis (LLMA), there are some studies proposing that the axis should be neutral [13]. It was stated that over or under-correction of the mechanical axis could cause clinical problems, which is why surgical planning is crucial [12]. Many techniques involving the intraoperative use of wire and fluoroscopy, advanced computer navigation systems, and assessment of the intended axis with imaging techniques in the preoperative period have been used to determine the degree of correction [34, 43]. There are various computer software programs to determine the proper correction angle of the mechanical axis, but these are not available to all orthopaedic surgeons [35, 39]. With the advent of the picture archiving and communications system (PACS), X-ray is one of the most commonly used and easily accessible technologies available to surgeons, allowing them to draw and obtain measurements. Two of the most common techniques used to detect LLMA in high tibial osteotomy (HTO) are the Miniaci and Dugdale techniques [9, 31].

However, to the best of our knowledge, no other study evaluated patients’ functional outcomes by comparing these two methods in medial open wedge high tibial osteotomy (MOWHTO), one of the most commonly performed osteotomies. This study aimed to compare the correction angles as determined both preoperatively and postoperatively by the Miniaci and Dugdale techniques in patients treated with MOWHTO and to evaluate their effect on the clinical outcomes.

## Methods

This study was designed as a retrospective cohort and approved by the local ethics committee. The data of 116 patients who underwent MOWHTO for osteoarthritis between January 2015 and November 2019 were retrieved from the hospital archive. Patients who were < 65 years old, patients with osteoarthritis only in the medial compartment of the knee (Kellgren Lawrence [KL] grade 2, 3 based on X-ray and magnetic resonance imaging -MRI) [24] who underwent a unilateral operation, patients with knee X-ray and MRI records, patients with stable joints and no knee contraction, patients with knee flexion > 100° and 5–15° varus, patients who were unresponsive to conservative treatment, and patients with a body mass index (BMI) < 30 kg/m^2^ were included in the study. Patients aged ≥ 65 years old, patients with osteoarthritis in regions other than the medial compartment on imaging studies (KL 2–4), patients who sustained a microfracture in the knee during arthroscopic intervention, patients who were operated on for meniscus or cruciate ligaments in the same session, patients who sustained a tibia plateau fracture with intraarticular extension during surgery, patients with findings of severe ligament failure on physical examination, patients with knee flexion < 100° and > 15° varus deformity, patients who have previously undergone a knee surgery, patients with an inflammatory disease (e.g., rheumatoid arthritis) and patients with a BMI ≥ 30 kg/m^2^ were excluded. In addition to clinical history and radiographs, patients with balance-related neurological and medical disorders, and those with joint osteoarthritis in lower extremities other than the knee were not included because of their influence on the Tinetti Gait and Balance Assessment (TGBA) and Western Ontario and McMaster Universities Osteoarthritis (WOMAC) scores utilized in this research [4, 33]. Therefore, 74 patients formed the study group.

### Evaluation of the clinical and radiological parameters

The patient’s clinical and demographic features included age, sex, operation side, BMI, bone union time, and degree of knee osteoarthritis. The correction angles for HTO were evaluated according to the Miniaci and Dugdale techniques on Weight-Bearing Scanograms (WBS). Angle measurements and surgeries were performed by two experienced orthopaedists (UA, MB), and patients were assigned to one of two groups according to the measurement technique to be used. Each surgeon used his angle measurement technique (UA; the Miniaci method-Group 1, MB; the Dugdale method-Group 2) for operation planning. The correction angle values at the patients’ last follow-up (5th year) were evaluated by the same surgeon who took the preoperative measurements using the same technique. Measurement values in the valgus position were expressed as negative. Two independent orthopaedists experienced in performing osteotomies around the knee were also included in the study to increase the reliability of the measurements. Per the study design, radiographic measurements were obtained preoperatively and via images taken at the 5-year follow-up. Scanograms were taken with the patient in the standing position, at a distance of 2.7 m. In both measurement techniques, the Mikulicz point was considered the exact lateral side of the lateral tibial eminence of the knee [28].

The correction angles that were measured at the last follow-up were compared between the groups and evaluated alongside the BMI values. The patients’ pre- and post-operative functional statuses were evaluated with the TGBA and WOMAC scale, and pain was evaluated with the Visual Analogue Scale (VAS) [4, 23, 33]. Using the first month evaluations as a reference for intraclass correlation coefficient (ICC) comparisons, the correction angles were evaluated twice at one-month intervals using different angle measurement techniques by blinded surgeons and orthopaedists. Preoperatively measured correction angles and patient BMI values were classified, and their relationships with TGBA, WOMAC, and VAS scores were considered.

### Correction angle measurement techniques

**Fig. 1:**
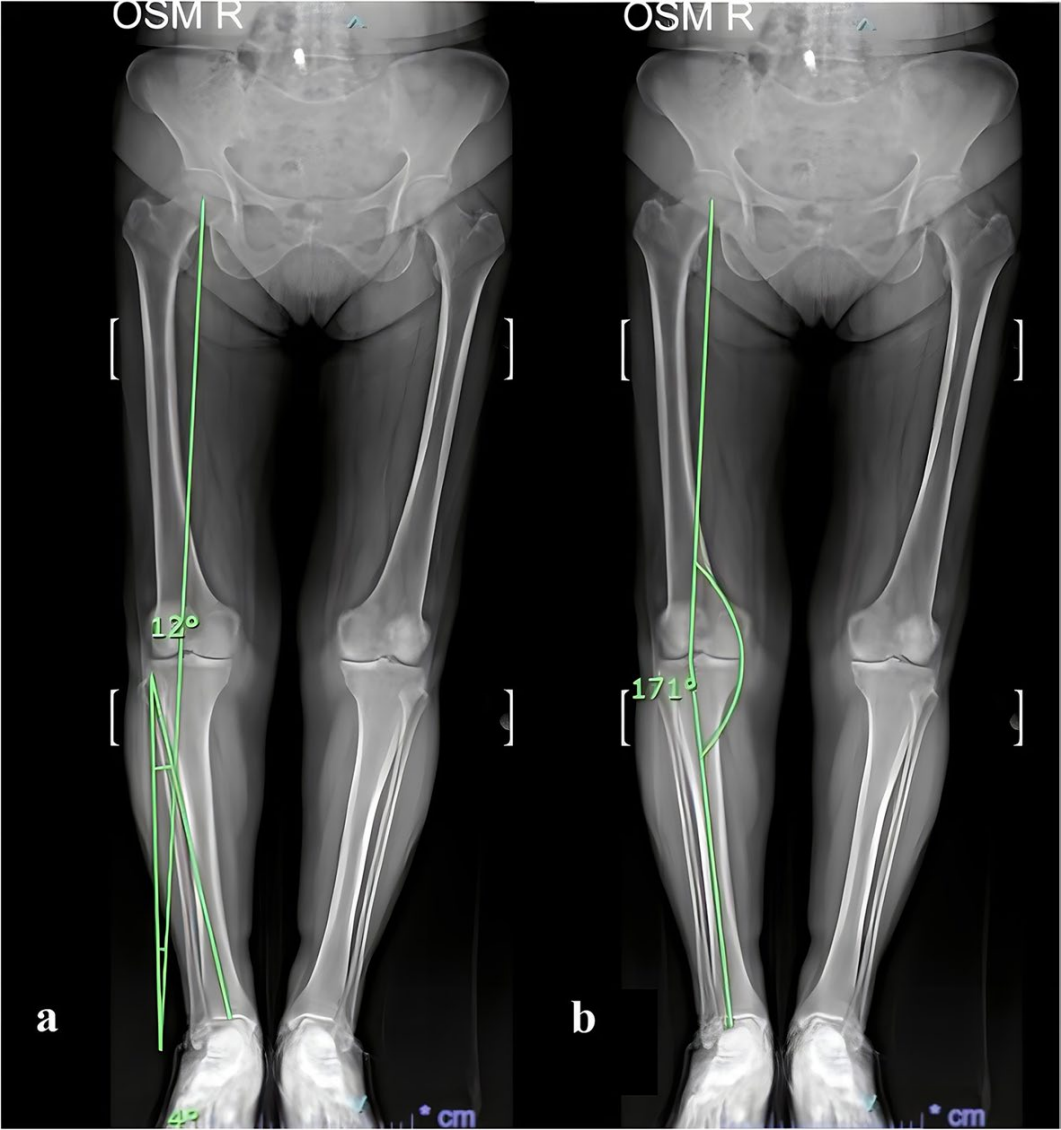
Determination of the correction angles in a patient's ipsilateral knee according to the Miniaci (**a**) and Dugdale (**b**) techniques (e.g., correction angle in **a**; as shown, in **b**; 180°—displayed angle value)

The Miniaci technique [31]: the planned weight-bearing line, known as the first line, passes through the predetermined goal point of the tibial plateau and extends from the hip joint's centre to the anticipated new centre of the ankle joint. The second line connects the centre of the ankle joint to the osteotomy hinge point. The third line connects the osteotomy hinge point to the anticipated new centre of the ankle joint. The intended correction angle is the angle formed by the second and the third line (Fig. 1a).

The Dugdale technique [9]: the first line extends from the hip joint's midpoint to the tibial plateau's preset target point (62% of the tibial plateau, according to Dugdale, measured from the medial border). The second line connects the ankle joint's midpoint to the tibial plateau's set target point. The intended correction angle is found by subtracting the angle formed by the first and second lines from 180° (Fig. 1b).

### Function and pain evaluation scales

The TGBA evaluates how stable and in balance a person feels while engaging in daily activities. It is made up of two separate parts, a gait subscale and a balance subscale (8 items and 12 points, 9 items and 16 points, respectively). The maximum score is 28, and the better the performance, the higher the score [33].

WOMAC Index: It has 24 questions, with a total score of 96 points, and is divided into three subcategories: function (0–68), stiffness (0–8), and discomfort (0–20). A better clinical result is indicated by a lower WOMAC score [4].

The VAS uses a 10 cm line with two end scores that signify 0 – no pain and 10 – severe pain to quantify the intensity of the discomfort during activities of daily living [23].

### Surgical technique

After spinal anaesthesia, all patients were prepared for ipsilateral iliac crest bone grafting in the supine position. After the tourniquet was inflated, the knee joint was arthroscopically evaluated first, and then the intraarticular distribution and osteoarthritis severity were determined. After arthroscopy, MOWHTO was performed as described elsewhere [3]. The breadth of the tibia at the osteotomy site and the required degree of correction were correlated using Hernigou's chart [20]. We sutured the long leg of the periosteum and appointed the superficial medial collateral ligament fibres only after the tourniquet was released. On the iliac wing and the osteotomy site, a Penrose drain was installed and removed the following day. It was demonstrated that suturing the inner collateral ligament fibres caused compression, which affected the desired correction [37].

### Statistical analysis

Study data were analysed using the SPSS (Statistical Package for Social Sciences) for Windows 25.0 (SPSS Inc., Chicago, IL) program. The normal distribution of data was evaluated with the Shapiro Wilk test. For data not normally distributed, the difference between quantitative data of two independent groups was compared with the Mann‒Whitney U test, more than two independent groups were compared with the Kruskal‒Wallis H test, and the corrected Bonferroni test was used to find the nonparametric group. Two dependent groups were compared with the Wilcoxon rank test, and chi-square analysis was used to detect the relationship between categorical variables. Consistency between measurements (intra/interobserver) was measured with the intraclass correlation (ICC) coefficient. *P* < 0.05 was considered statistically significant.

## Results

Seventy-four patients (62 females, 12 males) with a mean age of 53.7 ± 4.9 years were followed up for a mean of 67.4 ± 5.5 months. Between the two groups ( Group 1; n: 35, Group 2; n: 39), there was no significant difference in the clinical or demographic features (Table 1). While there was no significant difference between the two groups in terms of the preoperative correction angle (PCA) value or the last follow-up correction angle (LFCA) value, there was a significant difference in the correction angles within the groups (*p* < 0.05). There was no significant difference in the LFCA values concerning BMI between or within the groups (*p* > 0.05) (Table 2).

**Table 1:** Comparison of the clinical and demographic features between the two groups

		**Group 1**		**Group 2**		***P***
		mean ± sd		mean ± sd		
Age^a^, years		53,5 ± 4,9		52,8 ± 5,0		0,757
BMI^a^, kg/m^2^		26,8 ± 1,2		25,7 ± 1,4		0,791
Bone union time^a^, week		9,5 ± 1,2		10,1 ± 1,2		0,368
		**n**	**%**	**n**	**%**	
Sex^b^	Female	31	88,6	31	79,5	0,355
	Male	4	11,4	8	20,5	
Operation side^b^	Right	28	80,0	29	74,4	0,593
	Left	7	20,0	10	25,6	
Degree of knee osteoarthritis^b^	2	10	28,6	12	30,8	1,000
	3	25	71,4	27	69,2	

*BMI* Body Mass Index, *sd* Standard deviation, *n* number, *P* significant value

^a^Mann Whitney U test

^b^X^2^: chi square test

**Table 2:** Comparison of the PCA, LFCA, and LFCA grouped by BMI between or within the two groups

		**Group 1**		**Group 2**		***P***
		mean ± sd, °	95% CI	mean ± sd, °	95% CI	
**PCA**^**a**^		10,9 ± 1,5	10,3–11,4	9,8 ± 1,5	9,3–10,3	0,721
**LFCA**^**a**^		1,4 ± 1,1	1,0–1,8	1,3 ± 1,2	0,8–1,5	0,824
**P**		0,000*		0,000*		
**LFCA**^**b**^	**BMI < 28 kg/m**^**2**^	1,6 ± 1,1	1,1–2,1	1,3 ± 1,2	0,8–1,8	0,290
	**BMI ≥ 28 kg/m**^**2**^	0,8 ± 0,9	0,1–1,6	1,7 ± 0,9	1,0–2,3	0,088
**P**		0,097		0,279		

*PCA* Preoperative correction angle, *LFCA* Last follow-up correction angle, *CI* Confidence Interval, ° degree

*Significance

^a^Mann Whitney U test, Wilcoxon test

^b^Mann Whitney U test

The TGBA, WOMAC, and VAS scores were improved within the groups at the last follow-up evaluations compared to the preoperative period (*p* < 0.05). Although the TGBA and WOMAC scores were not different between the two groups in the preoperative period, they were worse in Group 2 than in Group 1 at the last follow-up (*p* < 0.05). The preoperative and last follow-up VAS scores were not significantly different between the groups (*p* > 0.05) (Table 3).

**Table 3:** Comparison of the TGBA, WOMAC, and VAS scores between or within the two groups

		**Group 1**		**Group 2**		***P***
		mean ± sd	95% CI	mean ± sd	95% CI	
**TGBA**	Preoperative	18,3 ± 0,9	18,0–18,6	17,6 ± 0,9	16,9–17,5	0,686
	Last follow-up	26,5 ± 1,1	26,1–26,9	23,6 ± 1,0	23,2–23,9	0,000*
	**P**	0,000*		0,000*		
**WOMAC**	Preoperative	86,6 ± 3,4	85,5–86,8	83,7 ± 2,6	83,5–85,3	0,084
	Last follow-up	20,3 ± 1,7	19,7–20,9	31,2 ± 2,3	30,4–31,9	0,000*
	**P**	0,000*		0,000*		
**VAS**	Preoperative	8,4 ± 0,9	8,0–8,7	7,5 ± 0,9	7,1–7,7	0,656
	Last follow-up	1,8 ± 0,8	1,5–2,1	1,9 ± 0,8	1,6–2,2	0,794
	**P**	0,000*		0,000*		

*TGBA* Tinetti Gait and Balance Assessment, *WOMAC* Western Ontario and McMaster Universities Osteoarthritis Index, *VAS* Visual Analog Scale

*Significance

Mann Whitney U test, Wilcoxon test

When measuring techniques were changed between the groups, the PCA and LFCA values were lower with the Dugdale technique in Group 1 patients but were higher with the Miniaci technique in Group 2 patients (*p* < 0.05). Intra- and inter-observer reliability was high in Group 1 when the PCA and LFCA were measured by using the Dugdale technique instead of the Miniaci technique; similarly, in Group 2 when the PCA and LFCA were measured by using the Miniaci technique instead of the Dugdale technique (Table 4).

**Table 4:** Evaluation of the groups with different correction angle measurement techniques and intra- and inter-observer reliability

		**MINIACI**		**DUGDALE**		***P***	**Intra-ICC**	**Inter-ICC**
		mean ± sd, °	95% CI	mean ± sd, °	95% CI			
**Group 1**	**PCA**	10,9 ± 1,5	10,3–11,4	8,0 ± 1,0	7,7–8,4	0,000*	0,898–0,945	0,932–0,967
	**LFCA**	1,4 ± 1,1	1,0–1,8	-1,1 ± 1,1	-1,5-(-0,7)	0,000*	0,936–0,987	0,971–0,991
**Group 2**	**PCA**	12,9 ± 1,5	12,4–13,3	9,8 ± 1,5	9,3–10,3	0,000*	0,941–0,974	0,946–0,982
	**LFCA**	3,7 ± 1,1	3,3–4,0	1,3 ± 1,2	0,8–1,5	0,000*	0,912–0,953	0,962–0,988

*PCA* Preoperative correction angle, *LFCA* Last follow-up correction angle, *ICC* Intra-class correlation coefficient

* Significance

Mann Whitney U test

There was no significant relationship between the PCA values and the TGBA and WOMAC scores at the last follow-up; however, the VAS scores that tended to decrease increased as the correction angle value increased (*p* < 0.05). There was no significant relationship between BMI and scores (*p* > 0.05) (Table 5).

**Table 5:** Relationship of PCA and BMI with TGBA, WOMAC, and VAS scores at the final follow-ups

		**TGBA**	**WOMAC**	**VAS**
		mean ± sd	mean ± sd	mean ± sd
**PCA**^**a**^	8 and 9, °	25,0 ± 1,5	27,1 ± 6,4	2,1 ± 1,2
	10 and 11, °	25,6 ± 1,4	26,1 ± 5,9	1,6 ± 0,6
	12–13-14, °	25,5 ± 1,3	25,4 ± 5,5	2,1 ± 0,7
	**P**	0,328	0,402	0,049*
**BMI**^**b**^	< 28 kg/m^2^	25,3 ± 1,3	26,1 ± 5,7	1,8 ± 0,7
	≥ 28 kg/m^2^	25,9 ± 1,5	25,8 ± 6,1	2,0 ± 0,9
	**P**	0,154	0,766	0,400

^a^Kruskal Wallis H test, Posthoc: Corrected Bonferroni, ^b^ Mann Whitney U test

* Significance

## Discussion

The most important findings of this study are that the Miniaci technique produced higher correction angle values, as high intra- and inter-rater reliability, with a simple viewing method compared to the Dugdale technique. The study's strength is confirmed in the fact that the patients who underwent open wedge HTO via the Miniaci technique had better functional statuses than those of patients with similar characteristics who underwent the procedure via the Dugdale method.

Although some studies suggest that computer navigation programs show the degrees of correction more accurately, this technology is expensive and not easily accessible to every surgeon [1, 21]. In most studies, researchers recommended avoiding under-correction (varus alignment) or extreme over-correction to achieve good therapeutic outcomes [15, 32]. Therefore, preoperative planning is a critical step in HTO. The course of degeneration changes depending on which part of the knee the mechanical axis passes through [11]. The Fujisawa point where the LLMA crosses the knee joint is located at 62.5% of the width of the knee joint from the medial side [15]. The other points that have been described in previous studies have been Dugdale (62%), Feller (58%), neutral (50%), or patient-specific points [2, 40, 41]. In this study, this point was determined as the lateral-to-lateral tibial eminence because we do not have a computer program that can show the measurements of the targeted location in the knee joint in detail. According to a study by Lee et al. [28], good surgical results can be obtained by simplifying the process of determining the location of the LLMA, ensuring that it passes through the lateral edge of the tibial intercondylar eminence.

It was reported that Miniaci’s method had high inter- and intra-observer reliability in measuring the PCA and the osteotomy gap [29]. Miniaci’s method was shown to be reliable regardless of the observer’s experience [11]. The cable approach under intraoperative fluoroscopic guidance was reported to be less accurate for controlling the resultant axis than the Miniaci method for measuring the gap [43]. According to Schröter et al. [36], using Dugdale's technique, there was a 0.8° difference between the planned and postoperative correction angles in the patients who underwent MOWHTO. Dugdale's method has comparably strong inter- and intra-rater reliability as Miniaci's method [5]. Three techniques [ the Miniaci Method, Navigation Assistance, and Patient-Specific Instrumentation (PSI)] were used to detect correction angles in patients who underwent open wedge HTO. After a 2-year evaluation, no method was superior to any other methods regarding correction values or clinical outcomes [45]. In another study of MOWHTO, if calibrated, PSI could provide better correction degrees and better clinical outcomes than conventional techniques [16]. Sivertsen et al. [38] used advanced statistics and found that in patients who underwent open and closed HTO, the correction angle values obtained via the Dugdale technique were lower than those obtained via the Miniaci technique. It was also stated that the Dugdale technique was an easier procedure than the Miniaci technique. Ribeiro et al. [34] compared the correction angle values acquired with a computer navigation system to those estimated with the Dugdale method. The correction angle obtained with the computer navigation system was approximately 19% larger than that predicted with the planned approach.

Although the simplicity of Dugdale's technique is appealing, the osteotomy's centre of rotation, situated some distance from the joint line's desired correction point, is ignored. This fact is considered when performing the Miniaci's method, which offers a planning process that more closely resembles the real operation’s process. The differences between techniques are more obvious in closed wedge osteotomy than in open wedge osteotomy. Compared to open wedge osteotomies, closed wedge osteotomies require that the centre of rotation be located away from the joint line, resulting in a further distance from the intended correction point [18, 38]. We believe that performing the same type of osteotomy for all patients would be beneficial for the comparison of techniques.

Yuan et al. [44] suggested that LLMA correction is correlated with functional improvement of the knee joints in patients undergoing MOWHTO. El-Azab et al. [10] observed that the under-correction group who underwent MOWHTO had a worse Lysholm score (used in knee function assessment). Parveen et al. [33] reported the high interrater reliability of the performance-oriented mobility assessment for patients with knee arthritis, indicating the utility of the examination in encouraging movement and improving balance in patients. In the present study, despite surgeries performed according to both measurement methods, improved functional and VAS scores indicate that MOWHTO is advantageous for patients with appropriate indications. Lower correction angle values with the Dugdale technique might have caused possible worsening of functional scores. However, satisfactory functional scores were still obtained in this group. As a result, surgeons who use either of these two methods in MOWHTO can help their patients achieve better functional outcomes.

In a study, high BMIs negatively affected knee joint function in patients who underwent MOWHTO. It was shown that patients with middle and low TGBA assessment scores did not have significantly different BMIs. The BMIs of patients with high scores were lower than those with low scores. They also reported that there was no link between BMI, VAS, and the Knee Society Score (KSS) and that there was a nonlinear relationship between BMI and TGBA evaluation scores [44]. Regarding the Oxford Knee Score, the scores of patients with a BMI < 25 kg/m^2^ and 25–30 kg/m^2^ were 3.5 and 1.8 points higher than those of patients with a BMI > 30 kg/m^2^, respectively [14]. In this study, we attribute our lack of difference between BMI and functional scores to the close follow-up of patients and the increasing influence of implant technology. The stability of the plate fixation is affected by the osteotomy gap since the plate must bear the weight of the patient. Therefore, preserving the osteotomy gap depends on the steadiness of the fixation. According to Hernigou et al. [19], patients treated with locking plates had better clinical outcomes than patients treated with traditional plates. There was also a decreased rate of correction loss in these individuals.

According to a research, patients with mid-level TGBA scores showed a greater degree of correction than those with low scores. On the other hand, patients with high scores had a smaller degree of correction than those with low scores [44]. In the same research, it was thought that this was due to the segmented effect of the correction degrees and the TGBA evaluation scores. In addition, the KSS scores were similar to the TGBA scores in terms of correction degrees. While there were no differences between the degrees of correction and the functional scores in the present study, while tending to decrease, the increase in VAS score with increasing PCA values leads us to believe that other factors should be considered in this surgery. In MOWHTO, Kamada et al. [22] reported that compared to the patients with a varus deformity less than 5°, the patients with a varus deformity greater than 5° were more likely to have under-correction and a significantly lower postoperative Lysholm score. The inadequate reproducibility of pre-intraoperative assessment tools, including navigation and fluoroscopy-based techniques, can be the cause of errors in coronal alignment correction [25, 30]. Soft tissue laxity surrounding the knee joint has been identified as a key determinant for corrective mistakes [26, 27]. In this study, there was no severe ligament damage in the knees in the preoperative and postoperative periods and no prominent correction loss in patients with severe osteoarthritis, suggesting that other factors might influence pain development.

The limitations of this study are its retrospective design, unbalanced sex distribution, and the inability to compare correction angles with advanced computer systems in terms of different implant types. Nevertheless, we believe that this study will provide surgeons with a different perspective on preoperative preparation by suggesting that MOWHTO, an effective surgical method, should be evaluated concerning several clinical parameters to determine the correction angle with simple methods in patients requiring HTO.

## Conclusion

In this study, the correction angle values obtained via the Miniaci method were higher than those obtained via the Dugdale method, which resulted in better functional scoring. Although all the patients who underwent MOWHTO showed satisfactory recovery, orthopaedic surgeons who use either of these two methods as a guide can help their patients achieve better functional outcomes postoperatively.
